# GAGE7B promotes tumor metastasis and growth via activating the p38δ/pMAPKAPK2/pHSP27 pathway in gastric cancer

**DOI:** 10.1186/s13046-019-1125-z

**Published:** 2019-03-11

**Authors:** Duan-Bo Shi, Ran-Ran Ma, Hui Zhang, Feng Hou, Xiang-Yu Guo, Peng Gao

**Affiliations:** 1 Department of Pathology, School of Medicine, Shandong University, Jinan, 250012 China; 2 Department of Pathology, Qilu Hospital, Shandong University, Jinan, 250012 China; 3 Department of Pathology, The Affiliated Hospital Of Qingdao University, Qingdao, 266071 China

**Keywords:** GAGE7B, Gastric cancer, Metastasis, Growth

## Abstract

**Background:**

Gastric cancer is the second most common cause of cancer-related mortality; thus, the mechanisms underlying tumor metastasis and growth in gastric cancer need to be extensively explored.

**Methods:**

Differentially expressed genes were examined in gastric cancer samples with lymph node metastasis (LNM) and without LNM using mRNA microarray and RT-qPCR. The effects of G antigen 7B (GAGE7B) on the metastasis, growth, and angiogenesis of gastric cancer were investigated in vitro and in vivo. GAGE7B protein expression was detected by immunohistochemical (IHC) analysis. Microarray, RT-qPCR, and western blot assays were performed to detect downstream target genes of GAGE7B. Dual-luciferase reporter and western blot assays were used to identify miRNAs that could negatively regulate GAGE7B.

**Results:**

GAGE7B was significantly overexpressed in samples with LNM. High expression levels of GAGE7B were associated with advanced clinical stage and poor patient survival. GAGE7B dramatically enhanced the metastasis, growth, and angiogenesis ability of gastric cancer. GAGE7B was further demonstrated to promote the progression of gastric cancer by activating the p38δ/pMAPKAPK2/pHSP27 pathway. However, the GAGE7B-induced p38δ/pMAPKAPK2/pHSP27 pathway was inactivated by miR-30c, as the expression levels of both GAGE7B and p38δ were found to be directly suppressed by miR-30c. Intriguingly, GAGE7B was found to be a ceRNA for p38δ, as it activated the p38δ/pMAPKAPK2/pHSP27 pathway by competitively binding miR-30c*.*

**Conclusions:**

GAGE7B may serve as a prognostic indicator in gastric cancer. GAGE7B significantly promotes gastric cancer progression by upregulating the p38δ/pMAPKAPK2/pHSP27 pathway, but it is negatively regulated by miR-30c. GAGE7B and miR-30c may be potential therapeutic targets in gastric cancer.

**Electronic supplementary material:**

The online version of this article (10.1186/s13046-019-1125-z) contains supplementary material, which is available to authorized users.

## Introduction

Gastric cancer is one of the most common malignancies and is the second most common cause of cancer-related mortality worldwide [1]. To date, surgery is still the most effective treatment for gastric cancer patients; however, the survival rate remains low, mainly due to tumor metastases [1, 2]. Therefore, it is vitally important to explore the mechanism(s) involved in the process of gastric cancer metastasis to elucidate potential targets for gastric cancer therapy.

In this study, differential expression analysis was performed using an mRNA microarray assay, comparing primary gastric cancer samples with and without lymph node metastasis (LNM). The results demonstrated that the GAGE genes were upregulated in the samples with LNM. The GAGE genes belong to the cancer testis antigen (CTA) gene family, which has a locus on chromosome X and consists of at least 16 genes that encode identical protein products. The genes encode proteins that are recognized by the immune system, thereby inducing an immune response [3, 4]. The protein is expressed in human cancers, but not in normal tissues, with the exception of immune-privileged germ cells [5, 6]. GAGE CTAs are thought to be potential immunotherapy targets in human cancers. Recent studies have shown that the expression of GAGE genes is correlated with poor prognoses in several human cancers including gastric cancer [7–9]. GAGE genes can also exert anti-apoptotic effects in human cancer cells [10]. GAGE12B, a member of the GAGE family, mediates human gastric carcinoma growth and metastasis [11]. However, the functions of the abnormally expressed GAGE genes in human cancer cells and the molecular mechanism mediating tumor initiation and progression in gastric cancer, are not well understood.

In the current study, the expression of G antigen 7B (GAGE7B), another member of the GAGE family, was found to be upregulated in gastric cancer. In addition, the functions of GAGE7B, promoting metastasis and the growth of gastric cancers, as well as the underlying mechanism related to its biological behavior, were investigated.

## Materials and methods

### Clinical samples

The clinical samples used in this study were obtained from Qilu Hospital of Shandong University and Shandong Provincial Hospital with approval from the Medical Ethics Committee of Shandong University (Ji’nan, Shandong, China). The fresh gastric cancer tissues were dissected from resected specimens by pathologists and then were immediately snap-frozen in liquid nitrogen for subsequent use. The formalin-fixed and paraffin-embedded samples were obtained from the Department of Pathology, Qilu Hospital, between 2005 and 2007. None of the patients contributing samples had received chemotherapy or radiotherapy. All samples were confirmed pathologically.

### Cell lines

The gastric cancer cell line AGS and human umbilical vein endothelial cells (HUVECs) were obtained from the American Type Culture Collection (ATCC, Manassas, VA, USA). BGC823 was purchased from the Chinese Academy of Sciences (Shanghai, China), and MKN45 was purchased from the Chinese Academy of Medical Sciences (Beijing, China). The cells were authenticated by DNA (STR) profiling and cultured in F12K (Gibco, Carlsbad, CA, USA) or RPMI-1640 medium (HyClone, Logan, UT, USA) supplemented with 10% fetal bovine serum (Gibco) and were incubated at 37 °C in a humidified incubator at 5% CO_2_.

### mRNA microarray analysis

Total RNA from fresh gastric cancer samples was extracted with TRIzol (Invitrogen, Carlsbad, CA, USA). RNA integrity and concentration were assessed after the RNA was extracted, prior to sample labeling. RNA labeling and hybridization on mRNA microarray chips was then carried out with a Human Gene Expression Microarray (Arraystar Human 8 × 60 K LncRNA + mRNA Microarray v2.0; Agilent Technologies). Sample labeling was carried out using an Agilent Quick Amp Labeling Kit (Agilent Technologies). Hybridization was subsequently performed using Agilent’s SureHyb Hybridization Chambers. The microarray images were quantified after detecting the hybridization signals using an Agilent DNA Microarray Scanner. The data were extracted using Agilent Feature Extraction software, and normalization was performed using the Agilent GeneSpring GX v12.1 software (in collaboration with the Kang Cheng Bio-Tech Corporation, Shanghai, China). A fold change > 2.0 and a *P* <  0.05 detected between the differentially expressed genes from the LNM samples and the non-LNM samples were considered to be significant.

### Real-time quantitative PCR

Real-time quantitative PCR (RT-qPCR) for analysis of mRNA and miRNA expression was performed as previously described [12, 13]. The primers are shown in Additional file 1: Table S3.

### Immunohistochemistry

Immunohistochemical (IHC) staining of the formalin-fixed and paraffin-embedded tissues was carried out using antibodies against GAGE7B (1:50, Proteintech, Wuhan, China), Ki-67 (1:100, DAKO, Glostrup, Denmark) and CD34 (1:100, DAKO), as previously described [14]. For GAGE7B, staining of the nucleus was considered positive after two pathologists independently scored the staining intensity and the corresponding percentage and agreed with one another’s analyses. The intensity was scored as 0 (negative staining), 1 (weak), 2 (moderate), and 3 (strong). The histoscore (Q) was calculated according to the following formula: Q = P_1_× 1 + P_2_× 2 + P_3_× 3 (P: percentage) [15]. The cases were divided into a low GAGE7B expression group and a high GAGE7B expression group based on the median of the Q values of GAGE7B expression (the median of the Q values: 85 × 1 + 0 × 2 + 0 × 3 = 85). The density of CD34 positive (CD34+) microvessels was calculated as previously described [14].

### Plasmid construction and dual-luciferase reporter assay

The coding sequence regions (CDS) of GAGE7B were synthesized and subcloned into a PcDNA3.1(+) vector (subsequently named PcDNA3.1-GAGE7B) by Sangon Biotech (Shanghai, China). The PcDNA3.1-GAGE7B or control plasmids were transfected into gastric cancer cells using Lipofectamine 2000 (Invitrogen), and the miR-30c (miR-30c-1-3P and miR-30c-2-3p) and negative control were transfected with X-tremgene (Roche, Applied Science, Indianapolis, IN, USA), according to the manufacturer’s instructions.

For the dual-luciferase reporter assay, the 3’UTR regions of GAGE7B and p38δ (MAPK13), containing the miR-30c binding sites, were synthesized and subcloned into pmirGLO or PcDNA3.1(+)-3’UTR vectors. The pmirGLO-3’UTR vectors and miR-30c or PcDNA3.1(+)-3’UTR were then co-transfected using Lipofectamine 2000. The relative expression of firefly luciferase was obtained after normalization to Renilla luciferase activity.

### Migration and invasion assays

The in vitro migration and invasion assays were performed as previously described [13].

### MTS and EDU assays

The proliferation ability of the transfected cells was examined using an MTS assay with a CellTiter 96 AQ_ueous_ One Solution Cell Proliferation Assay kit (Promega, San Luis Obispo, CA, USA) and an EdU assay with a Cell-Light TM EdU Apollo 567 in vitro kit (Ribobio, Guangzhou, China), according to the manufacturer’s instructions.

### Apoptosis assay

An Annexin V-FITC/PI Apoptosis Detection Kit (BestBio, Shanghai, China) was used to detect apoptosis of transfected cells according to the manufacturer’s instructions. Briefly, the cells were trypsinized, collected, and washed twice with cold 1 × PBS. The washed cells were suspended in 400 μl 1 × binding buffer and then stained with 5 μl Annexin V-FITC for 15 min at 4 °C in the dark. The cells were subsequently stained with 10 μl propidium iodide (PI) for 10 min at 4 °C in the dark. The stained cells were then analyzed for early and late apoptosis via flow cytometry.

### Western blot assay

Western blotting was performed using antibodies against GAGE7B (1:200, Proteintech, Wuhan, China), p38δ, phospho-MAPKAPK2 (pMAPKAPK2), and phospho-HSP27 (pHsp27) (1:1000, CST, Danvers, MA, USA), as previously described [13].

### RNA-binding protein immunoprecipitation (RIP) assay

An EZ Magna RIP kit (Millipore, Billerica, MA, USA) was used to perform the RIP assay. In brief, gastric cancer cells were lysed using RIP lysis buffer. The extract was then incubated with RIP buffer containing magnetic beads conjugated with human AGO2 antibody or negative control IgG (Millipore, USA). The beads were washed, and the RNA in the sample was isolated after the protein was digested via incubation with Proteinase K. The purified RNA was subjected to RT-qPCR assay.

### Orthotopic tumor model

The BGC823 cells were first transfected with lentivirus vector LV-GV416-GAGE7B or LV-GV416-negative control (Genechem, Shanghai, China), and 1.5 × 10^6^ transfected cells were then harvested and injected into the left axillary fossa of Nu/Nu mice in order to detect the stromal invasion ability of the cells. For the hematogenous metastasis experiment, 1.5 × 10^6^ transfected cells were injected into the lateral tail vein of the mice. An in vivo Carestream Molecular Imaging system (Carestream Health, Inc., New York, NY, USA) was used to detect the xenografted tumors in living mice. The tumor volume (V) was calculated as V = *a* × *b*^2^ × 0.5, where *a* is the long axis and b is the short axis of the tumor nodules. The mice were euthanized 5 weeks after injection, and the tumor nodules, lungs, and liver were collected for further assessment. The liver and lungs were embedded in paraffin, cut into 4-μm sections, and stained with hematoxylin-eosin. For analysis of liver and lung metastases, five fields were randomly chosen on each slice, and the number of metastases was counted in each field.

### Statistical analysis

Prism 5 software (GraphPad Software, San Diego, CA, USA) was used for statistical analyses. The data are expressed as the median ± SE. The Student *t-*test (two-sided) was used for difference analyses between two groups; Kaplan-Meier survival analysis and the log-rank test were used for patient survival analysis. The correlation between miR-30c and GAGE7B was calculated using Spearman’s correlation. Values of *P* less than 0.05 were considered statistically significant.

## Results

### The expression of GAGE7B in gastric cancer tissues and correlation with clinicopathological features

**Fig. 1:**
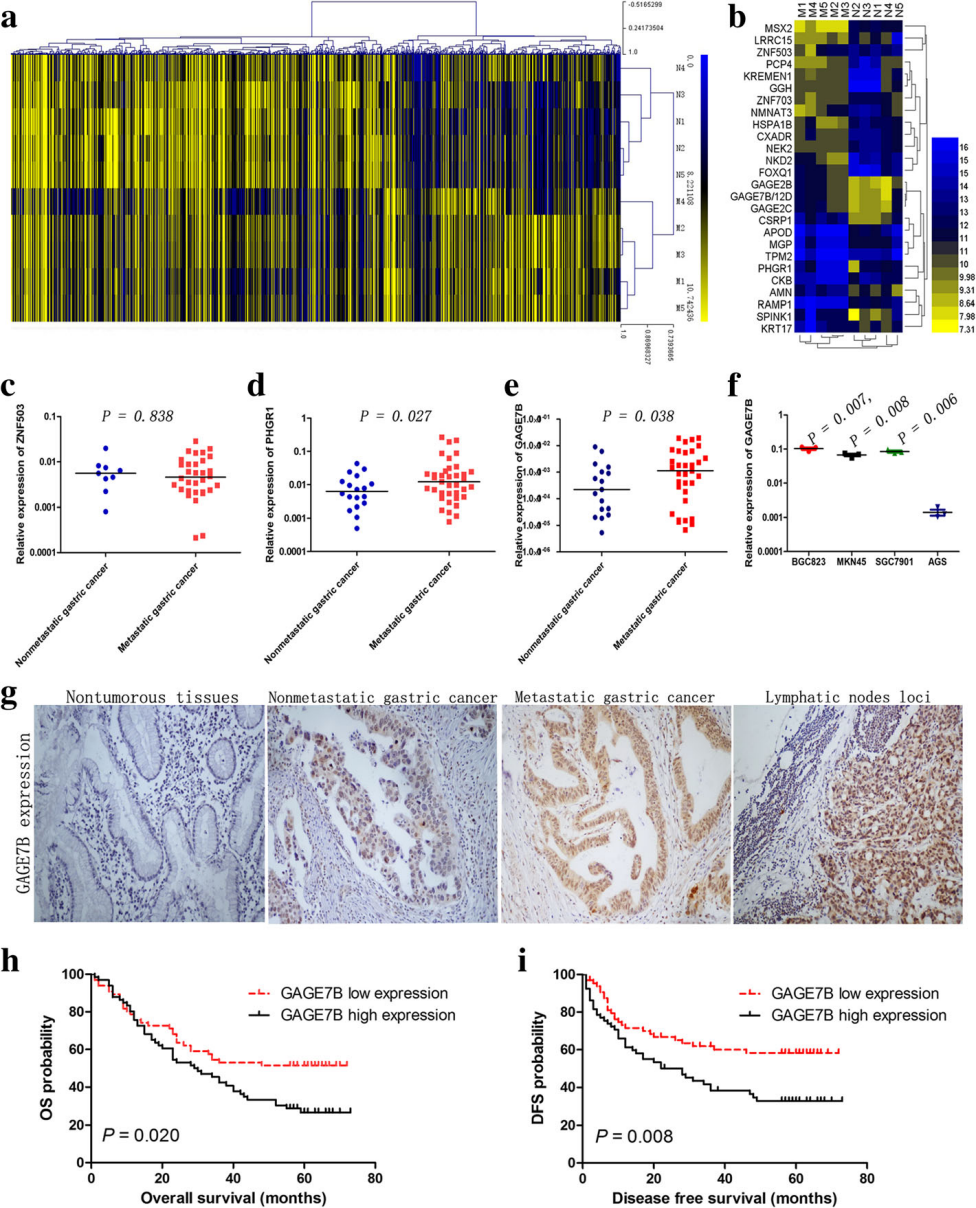
The expression of GAGE7B in gastric cancer tissues and its correlation with clinicopathological features. a and b. Microarray data showed 700 downregualted genes and 450 upregualted genes (fold-change ≧2, *P <  0.05*) between metastatic cancer tissues (M group) and nonmetastatic cancer tissues (N group) (**a**). The differentially expressed genes were further identified according to the fold-change (≧4), raw value (≧300) and *P* value (*P < 0.05*). The results showed that 13 genes were significantly downregulated and 13 genes including GAGE7B were significantly upregulated in metastatic tissues (**b**). **c-e**. The result of RT-qPCR confirmed that the expressions of PHGR1 and GAGE7B, but not ZNF503, were upregulated in metastatic cancer tissues, compared with nonmetastatic cancer tisssues (*t-test, P = 0.838, P = 0.027, P = 0.038*, respectively). **f**. GAGE7B mRNA expression was significantly increased in poorly differentiated gastric cancer cell lines BGC823, MKN45 and SGC7901, compared with the well differentiated cell line AGS (*t-test, P = 0.007, P = 0.008 and P = 0.006*, respectively). **g**. The protein expression of GAGE7B was demonstrated to be negative in nontumorous tissues. In contrast, positive staining of GAGE7B protein was observed in tumor samples and GAGE7B protein expression was increased in metastatic gastric cancer tissues in comparison with nonmetastatic cancer tissues (× 200). The GAGE7B expression was further increased in metastatic loci, compared with the primary lesions. **h** and **i**. In Kaplan-Meier survival analysis and Log-rank Test, the patients with a higher GAGE7B expression had shorter OS (e, *P = 0.020*) and DFS than those with lower expression (f, *P = 0.008*)

A microarray assay was performed to identify the genes differentially expressed between metastatic and nonmetastatic gastric cancer tissues. Hundreds of differentially expressed genes were found (GEO: GSE72307, Fig. 1a). Upon further analysis, a total of 26 genes were identified, which were considered to be the most aberrantly expressed genes (Fig. 1b). The genes that had not been previously well-investigated, among the 26 genes, including PHGR1, ZNF503, and GAGE7B were selected for further study. The expression levels of PHGR1 and GAGE7B were found to be significantly upregulated in metastatic compared with nonmetastatic tissues (Fig. 1c-e). Moreover, the expression of GAGE7B was higher in the poorly differentiated gastric cancer cell lines BGC823, MKN45, and SGC7901, compared with the well differentiated AGS cell line (Fig. 1f). GAGE7B expression was also upregulated in the samples from advanced stage cancers (stages III-IV, 7th TNM stage [16]) compared with samples from earlier stages (I/II) of gastric cancer (Table 1).

**Table 1:** Clinicopathologic characteristics of gastric cancers associated with GAGE7B mRNA expression

Variables	GAGE7B		
	*NO.*	Median	*P* Value
Age (y)			
≤ 60	14	0.096	*0.239*
> 60	36	0.064	
Gender			
Male	39	0.058	*0.322*
Female	11	0.157	
Tumour size (mm)			
<50	18	0.123	*0.331*
≥ 50	32	0.048	
^7th^ TNM stage			
I / II	23	0.045	*0.034*
III /IV	27	0.134	
Lymph node metastasis			
Yes	33	0.114	*0.038*
No	17	0.022	
WHO histological classification			
Well/Moderately differentiated	18	0.051	*0.551*
Poorly differentiated	32	0.099	
Lauren’s classification			
Intestinal type	25	0.045	*0.903*
Diffuse type	25	0.134	

Since antibodies for PHGR1 are not available at present, only the expression of GAGE7B was examined by IHC. The results showed that there was no GAGE7B protein expression in 16 samples of nontumorous gastric epithelium. In contrast, GAGE7B staining was positive in 117 of 132 tumor samples. The histoscore of GAGE7B protein expression was much higher in metastatic samples than that in nonmetastatic samples and was even higher in the lymph node metastatic loci samples compared with that in the primary lesions (Fig. 1g). In addition, the histoscore was higher in advanced-stage gastric cancer samples and was also higher in samples from older patients (> 60 y) (Table 2).

**Table 2:** Clinicopathologic characteristics of gastric cancers associated with GAGE7B protein expression

Variables	GAGE7B		
	NO.	Median	*P-* Value
Age (y)			
< 60	61	80.0	0.025
≧ 60	71	90.0	
Gender			
Male	110	82.5	0.424
Female	22	115.0	
Tumour size (mm)			
<50	67	80.0	0.495
≥ 50	65	90.0	
^7th^ TNM stage			
I / II	46	62.5	0.009
III /IV	86	90.0	
Lymph node metastasis			
Yes	90	90.0	0.012
No	42	70.0	
WHO histological classification			
Well/Moderately differentiated	40	82.5	0.771
Poorly differentiated	92	85.0	
Lauren’s classification			
Intestinal type	56	82.5	0.918
Diffuse type	76	85.0	
Lymph node loci analysis			
Lymph node loci	74	190.0	< 0.001
Paired primary gastric cancer	74	107.5	

Patients with higher GAGE7B expression had a shorter OS and DFS than patients with lower GAGE7B expression (Fig. 1h and i). The multivariate analysis indicated that clinical stage and tumor size were independent unfavorable predictors for OS and DFS (Additional file 1: Table S1).

### GAGE7B enhances the invasion and metastatic ability of gastric cancer cells

**Fig. 2:**
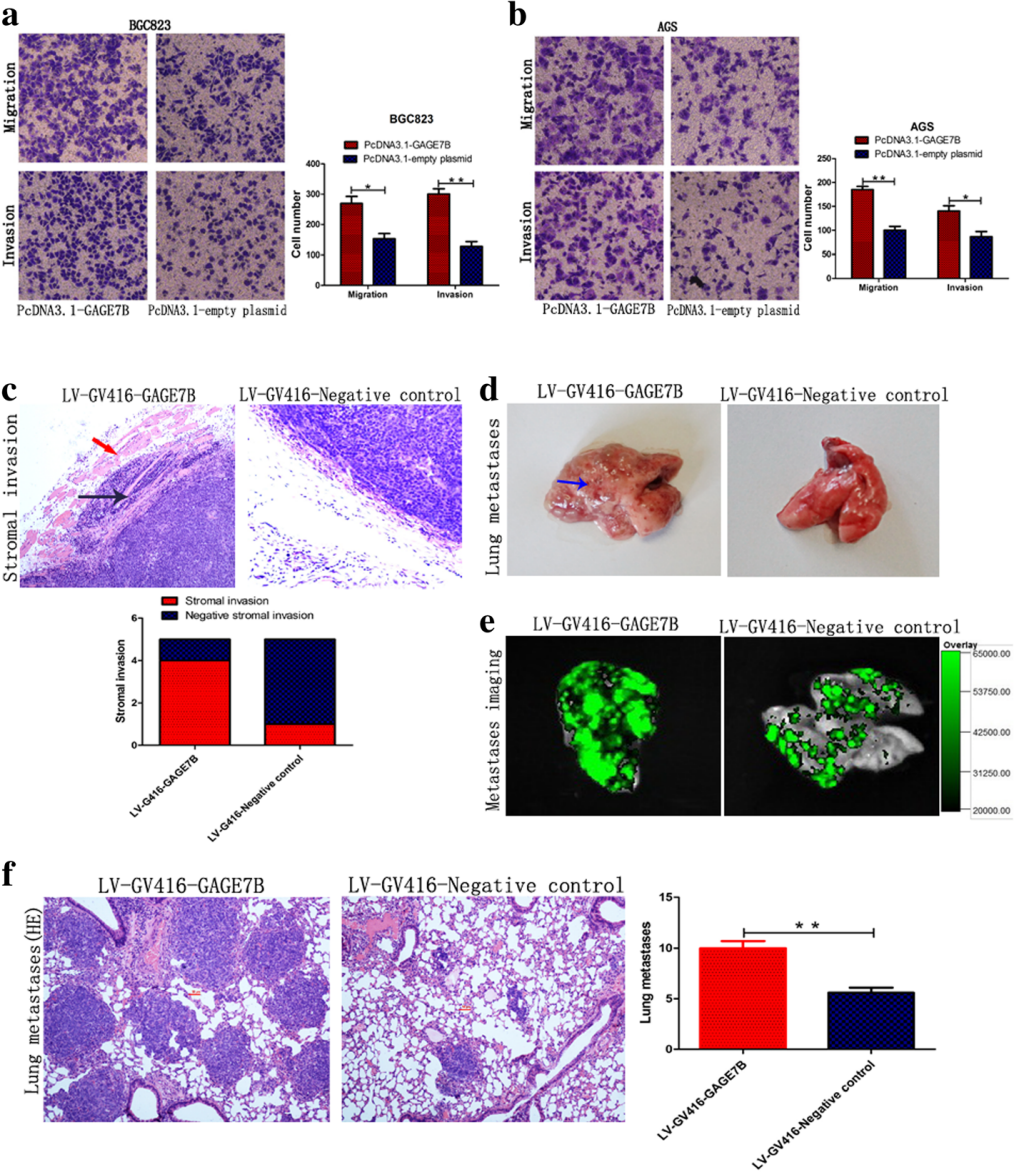
GAGE7B enhances the invasion and metastatic ability of gastric cancer cells. **a** and **b**. The transwell assay was performed in vitro and the results showed that the migration and invasion ability of BGC823 and AGS cells was significantly enhanced after GAGE7B was overexpressed (t-test, **P < 0.01, *P < 0.05). **c-f** The influence of GAGE7B on gastric cancer cells’ invasion and metastasis was investigated in vivo. The BGC823 cells were transfected with LV-GV416-GAGE7B or LV-GV416-Negative control with GFP (green fluorescent protein) as a marker protein and then were injected into the left axillary fossa or tail vein of the mice. More stromal invasion incidents of the tumor nodules were observed in GAGE7B group (4/5), compared with negative control group (1/5) (× 100). The red arrow showed that the GAGE7B overexpressing cells invaded into the stroma, the black arrow showed the muscular tissues and the blue arrow showed the lung metastases (**c**). Metastases could be observed on the surface of the lungs (**d**). In vivo imaging showed that the metastases were derived from GFP marked gastric cancer cells (**e**). HE staining showed more lung metastases in GAGE7B group (**f**) (× 100, t-test, **P < 0.01)

To explore the function of GAGE7B in gastric cancer, we transiently overexpressed GAGE7B in gastric cancer cells (Fig. 4b and Additional file 1: Figure S1a). In the subsequent Transwell assay, enhanced migration and invasion abilities were observed in the GAGE7B-overexpressing cell lines (Fig. 2a, b, and Additional file 1: Figure S1b). In contrast, knockdown of GAGE7B decreased the migration and invasion ability of gastric cancer cells (Additional file 1: Figure S1c, d, e and f). To investigate the role of GAGE7B on the metastatic capability of cancer cells further in an in vivo system, we first transfected BGC823 cells with LV-GV416-GAGE7B or LV-GV416-negative control. The LV-GV416-GAGE7B-transfected cells were found to express GAGE7B stably (Additional file 1: Figure S2). The transfected cells were then injected subcutaneously into the left axillary fossa of mice in order to determine the invasion ability of the cells. Five weeks after injection, the incidence of stromal invasion of the primary tumor in the GAGE7B group (4/5) was significantly higher than in the negative control group (1/5), suggesting that GAGE7B enhanced the invasion ability of cancer cells in vivo (Fig. 2c). As expected, more metastatic loci were observed in the lungs of the mice injected with LV-GV416-GAGE7B transfected cells than that in the lungs of the mice in the negative control group (Fig. 2d-f) in hematogenous metastasis experiments.

### GAGE7B promotes tumor growth in vivo

**Fig. 3:**
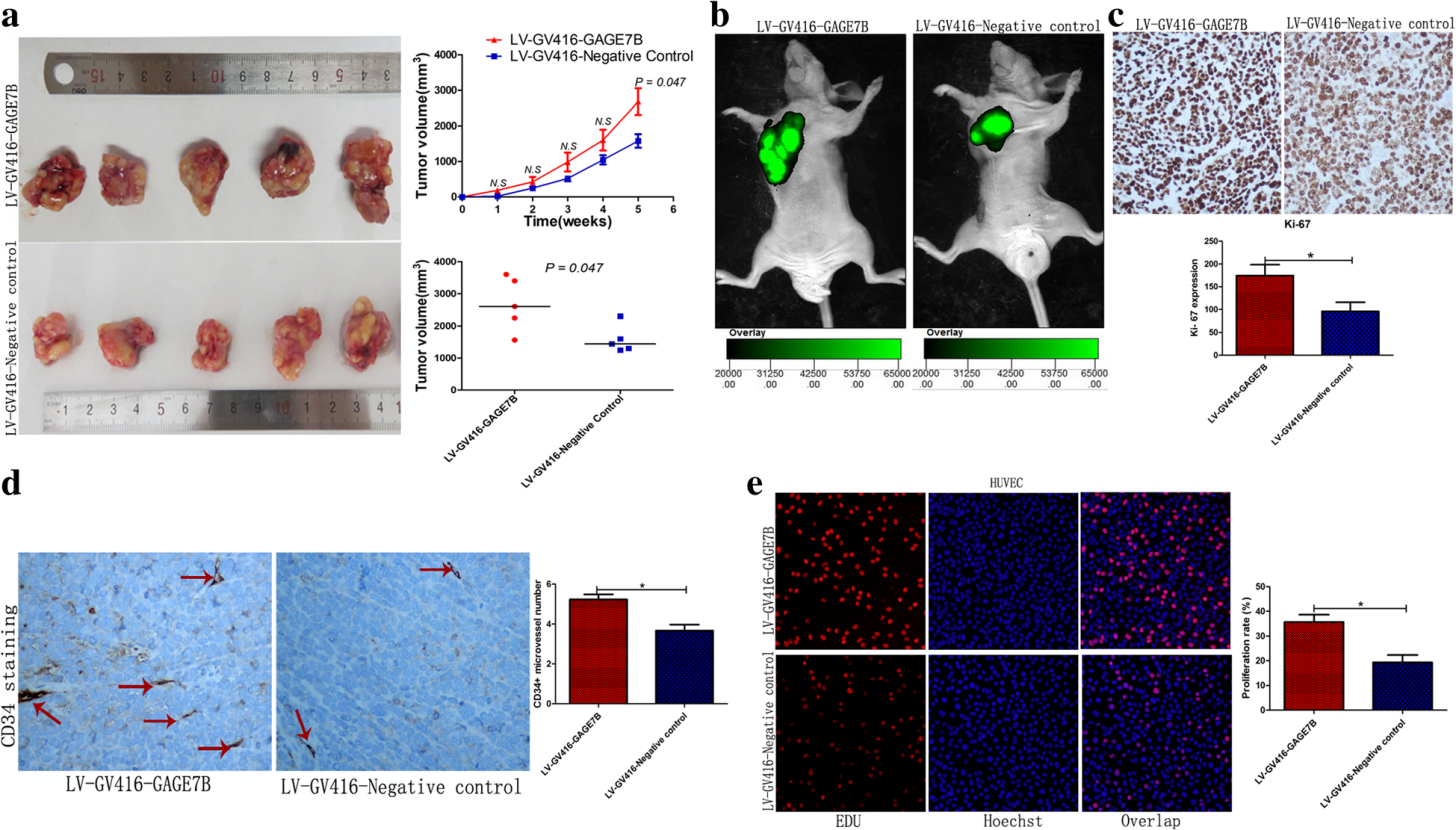
GAGE7B promotes tumor growth and angiogenesis in gastric cancer. **a-c** GAGE7B promoted tumor growth in vivo. The tumor nodules derived from GAGE7B overexpressing cells grew faster during the experiment, compared with that in negative control group. The mean tumor volume of the tumor nodules in GAGE7B group was 2682 ± 375 mm^3^, while it was 1578 ± 191 mm^3^ in negative control group at the end of 5th week (t-test, *P < 0.01) (**a** and **b**). Consistently, ki-67 staining verified that GAGE7B promoted gastric cancer growth in vivo (× 100, t-test, *P < 0.01) (**c**). **d**. GAGE7B induced angiogenesis in vivo. IHC was performed for CD34 staining in the tumor nodules derived from the mice. The analysis suggested that the CD34+ microvessels were more in LV-GV416-GAGE7B group than that in negative control group (× 400, t-test, *P < 0.05). **e**. The supernatant of LV-GV416-GAGE7B and LV-GV416-Negative control transfected gastric cancer cells was collected and then HUVEC cells were cultured with the collected supernatant for 24 h. The result of EDU assay suggested that the proliferation ability of HUVEC was enhanced in GAGE7B group (t-test, *P < 0.05)

Though GAGE7B had no significant effect on the proliferation ability of AGS and BGC823 cells in vitro, as shown by MTS, CCK8, and EdU assays, or on cancer cell apoptosis, as shown by the flow cytometry assay (Additional file 1: Figure S3 and S4), GAGE7B did significantly influence tumor growth in vivo. The subcutaneous tumor nodules derived from the LV-GV416-GAGE7B mice grew faster than those derived from the negative control group (Fig. 3a and b). In addition, the proliferation index (Ki-67 expression) was higher in GAGE7B-overexpressing cells from tumor nodules than in negative control cells (Fig. 3c).

### GAGE7B stimulates angiogenesis in gastric cancer

It is well-known that angiogenesis is essential for tumor growth and metastasis in human cancer. Given that GAGE7B promotes tumor metastasis and growth in gastric cancer, it may also be that GAGE7B contributes to gastric cancer progression, at least partially, by stimulating angiogenesis. Microvessel density analysis in the subcutaneous tumor nodules showed that the number of the CD34+ microvessels in the GAGE7B group was higher than in the negative control group (Fig. 3d). The association between microvessels and GAGE7B expression was further analyzed in human gastric cancer tissue. The number of CD34+ microvessels in the high-GAGE7B-expression group was significantly higher than in the low-GAGE7B-expression group (Additional file 1: Figure S6). In addition, the proliferation ability of HUVECs was significantly enhanced after these cells were cultured with the supernatant from LV-GV416-GAGE7B transfected cells (Fig. 3e).

### GAGE7B promotes the progression of gastric cancer via activating the p38δ/pMAPKAPK2/pHSP27 pathway

**Fig. 4:**
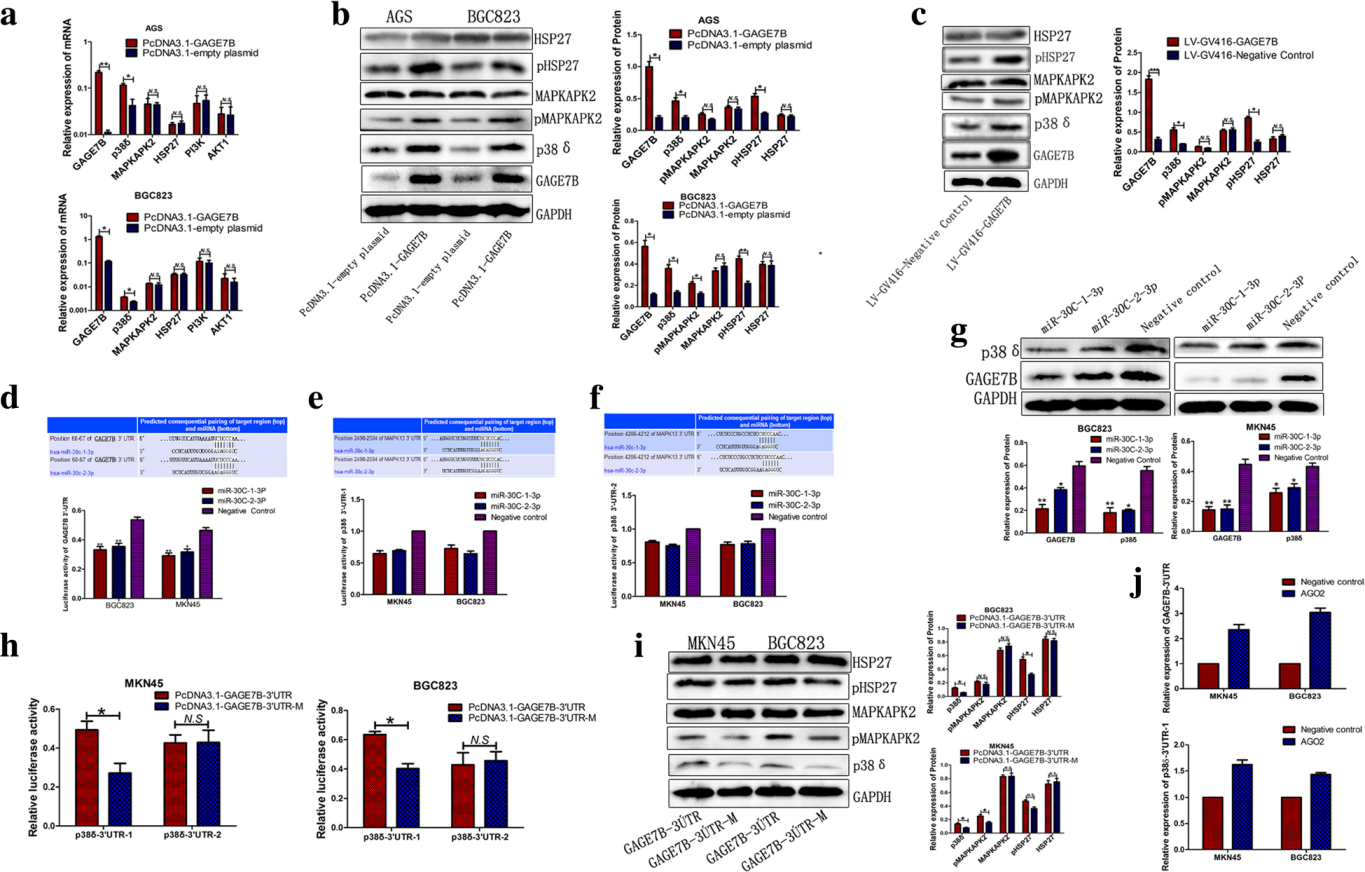
GAGE7B activates p38δ/ pMAPKAPK2/ pHSP27 pathway. **a** The upregualted mRNA expressions of p38δ in AGS and BGC823 cells were validated by RT-qPCR (t-test, **P < 0.01, *P < 0.05). However, the effect of GAGE7B on the expressions of MAPKAPK2, HSP27, PI3K and AKT1 was not significant (*t-test, P > 0.05*). **b** The analysis revealed that the protein expressions of p38δ, pMAPKAPK2, and pHSP27 were significantly upregualted by GAGE7B in AGS or BGC823 cells in Western-blot assay (t-test, **P < 0.01, *P < 0.05). Though, the upregualtion of pMAPKAPK2 was not significant, its expression was upregulated by 43.3% in AGS cells. However, the total protein expressions of MAPKAPK2 and HSP27 were not influenced by GAGE7B (*t-test, P > 0.05*). **c** The expressions of the genes of p38δ/ pMAPKAPK2/ pHSP27 pathway, including p38δ and pHSP27, were significantly upregulated, with the upregulation of pMAPKAPK2 by 39.8%, by GAGE7B, indicating that GAGE7B activated p38δ/ pMAPKAPK2/ pHSP27 pathway in vivo (*t-test,* *P < 0.05,**P < 0.01,***P < 0.001). **d-g** The relative luciferase activity of GAGE7B pmirGLO-3′ UTR vector was significantly decreased by miR-30c in two gastric cancer cell lines (d) (t-test, **P < 0.01, *P < 0.05). In addition, two binding sites of miR-30c in p38δ (MAPK13)-3′ UTR were predicted, and the relative luciferase activity of p38δ-3′ UTR-1 was reduced by miR-30c-1-3p and miR-30c-2-3p by 35.3 and 30.9% in MKN45 cells, and was reduced by 27.4 and 35.6% in BGC823 cells. While, the relative luciferase activity of p38δ-3′ UTR-2 was reduced by miR-30c-1-3p and miR-30c-2-3p by 19.4 and 24.8% in MKN45 cells, and was reduced by 23.1 and 21.8% in BGC823 cells (e and f). GAGE7B and p38δ protein expressions were dramatically reduced by miR-30c in Western-blot assay (**g**) (t-test, **P < 0.01, *P < 0.05). h and i. The relative luciferase activity of p38δ-3ÚTR-1, but not p38δ-3ÚTR-2, was enhanced after the transfection of GAGE7B-3’UTR in gastric cancer cells, compared with that in GAGE7B-3’UTR-M group (t-test, *P < 0.05) (**h**). The upregulation of p38δ protein by GAGE7B-3’UTR was detected in Western-blot assay. And the upregulation of p38δ, pMAPKAPK2 and pHSP27, but not MAPKAPK2 or HSP27, by GAGE7B-3’UTR was also revealed (t-test, *P < 0.05) (**i**). **j** The result of RIP assay showed that GAGE7B-3’UTR was enriched in the AGO2 pellet relative to negative control IgG by 2.8 fold and 2.5 fold in BGC823 and AGS cells, respectively. Simultaneously, p38δ-3’UTR-1 was enriched by 1.6 fold in MKN45 cells and 1.4 fold in BGC823 cells

An mRNA microarray assay was performed with gastric cancer cells to identify the pathways that are regulated by GAGE7B. The regulated pathways related to tumor metastasis, growth, or angiogenesis were selected for further analysis. The results showed that the expression levels of the genes that involved in p38/pMAPKAPK2/pHSP27 and PI3K/AKT pathways were significantly upregulated by GAGE7B (Additional file 1: Figure S5). The upregulated mRNA expression levels of the genes were then validated by RT-qPCR (Fig. 4a). The increased protein expression levels of p38δ, pMAPKAPK2, and pHSP27 (which are related to tumor growth, and metastasis) were further validated by western blot assay (Fig. 4b and c).

### The GAGE7B-induced p38δ/pMAPKAPK2/pHSP27 pathway is negatively regulated by miR-30c

**Fig. 5:**
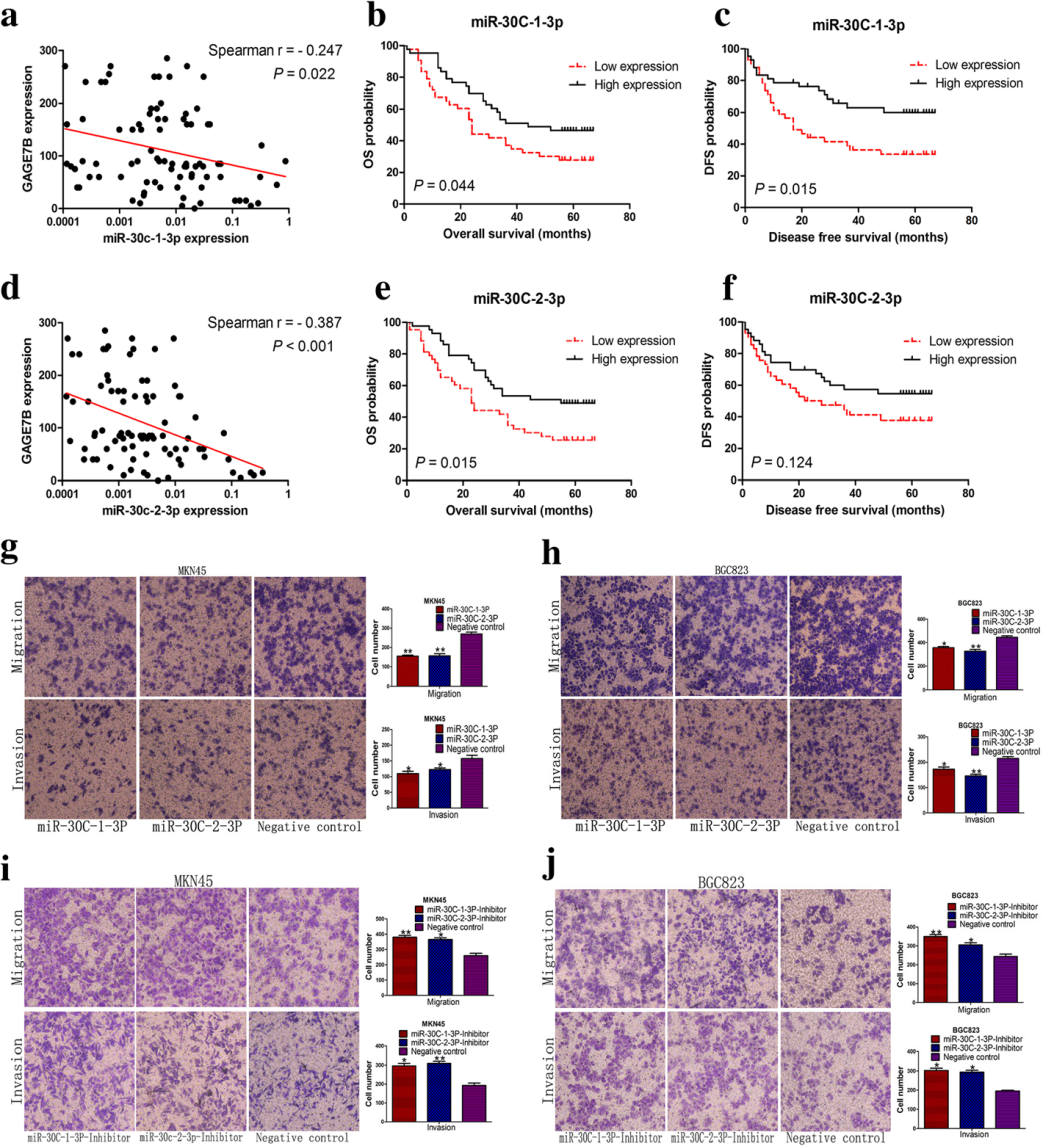
miR-30c inhibits the migration and invasion ability of gastric cancer cells and is associated with patients’ survival. **a** and **d** Correlation analysis showed that both miR-30c-1-3p and miR-30c-2-3p expressions were inversely correlated with GAGE7B expression (*Spearman r = − 0.247* and *- 0.387*, respectively). **b** and **c** In patients’ survival analysis, low expression of miR-30c-1-3p was associated with patients’ poor OS (**b**) and DFS (**c**) (*Log-rank test, P = 0.044* and *P = 0.015,* respectively). e and f. The association between miR-30c-2-3p expression and patients’ OS (**e**) and DFS (**f**) was simultaneously analyzed and the results suggested that low expression of miR-30c-2-3p was also associated with poor patients’ OS (*Log-rank test, P = 0.015*). **g** and **h** The migration and invasion ability of MKN45 and BGC823 cells was significantly inhibited upon overexpression of miR-30c-1-3p and miR-30c-2-3p (**g** and **h**). (t-test, **P < 0.01, *P < 0.05). i and j. The enhanced migration and invasion ability of MKN45 (**i**) and BGC823 (**j**) cells was observed in miR-30c-1-3p or miR-30c-2-3p knockdown group. (t-test, *P < 0.05,**P < 0.01)

miRNAs have been demonstrated to play important roles in human cancers by negatively regulating their target genes. The target miRNAs of GAGE7B were first predicted using software (Targetscan, Pictar, and Miranda). miR-887-5p, miR-320b, miR-590-3p, miR-30c-1-3p, and miR-30c-2-3p were then selected as the potential miRNAs targeting GAGE7B. A dual-luciferase reporter assay subsequently revealed that miR-30c-1-3p and miR-30c-2-3p significantly suppressed GAGE7B expression by binding to the sequences at 61 bp–67 bp of the GAGE7B-3’UTR (Fig. 4d), while the other three miRNAs had no significant effect on GAGE7B expression (Additional file 1: Figure S7a, b and c). The inhibition of GAGE7B protein expression by miR-30c-1-3p and miR-30c-2-3p was further confirmed by western blot assay (Fig. 4g). When the binding site of miR-30c-1-3p and miR-30c-2-3p in the GAGE7B-3’UTR was mutated, the regulation of GAGE7B by miR-30c-1-3p and miR-30c-2-3p was attenuated (Additional file 1: Figure S7d). Of note, miR-30c-1-3p and miR-30c-2-3p were both inversely correlated with GAGE7B expression in gastric cancer samples (Fig. 5a and d), and miR-30c-1-3p expression was also found to be downregulated in the samples with LNM (Additional file 1: Table S2). In addition, low expression levels of miR-30c-1-3p and miR-30c-2-3p were associated with poor OS in patients (Fig. 5b and e). Low expression of miR-30c-1-3p was also correlated with poor DFS in patients (Fig. 5c and f).

In addition, p38δ was predicted to be another target gene of miR-30c. The results of the dual-luciferase reporter assay and western blot assay showed that overexpression of miR-30c not only suppressed GAGE7B expression but also significantly suppressed p38δ expression (Fig. 4e-g, Additional file 1: Figure S7e and S7f).

Ectopic expression of miR-30c inhibited the migration and invasion ability of gastric cancer cells (Fig. 5g and h). In contrast, downregulation of miR-30c enhanced the migration and invasion ability of cancer cells (Fig. 5i and j). However, miR-30c had no significant influence on the proliferation ability of gastric cancer cells in vitro (Additional file 1: Figure S8, S9 and S10).

### GAGE7B is a ceRNA for p38δ

It has been reported that mRNAs, which harbor miRNA-response elements (MREs) in the 3’UTRs, could function as competing endogenous RNAs (ceRNAs) by competitively binding miRNAs [17]. Thus, it was of great interest to investigate whether GAGE7B mRNA could be a ceRNA for p38δ by competing for miR-30c. p38δ expression was significantly upregulated in gastric cancer cells overexpressing GAGE7B-3’UTR, compared with that in GAGE7B-3’UTR-M (which had a miR-30c binding site mutation) group in dual-luciferase and western blot assays (Fig. 4h and i). In addition, the expression levels of p38δ, pMAPKAPK2, and pHSP27 were also significantly upregulated in at least one of gastric cancer cell lines in GAGE7B-3’UTR group in the western blot assay (Fig. 4i). It is well known that the RNA-induced silencing complex (RISC) containing AGO2 is necessary for miRNA-mRNA binding. As we expected, the RIP assay showed that GAGE7B-3’UTR was enriched in AGO2 pellets relative to the negative control (Fig. 4j); p38δ-3’UTR-1 was also enriched in AGO2 pellets relative to negative controls in gastric cancer, though not as much as GAGE7B-3’UTR was (Fig. 4j).

## Discussion

The results of the current study provide insights into the expression and function of GAGE7B in gastric cancer. To the best of our knowledge, this is the first time that the mechanisms underlying the roles of GAGE7B in gastric cancer have been investigated. In this study, a new mechanism for the regulation of GAGE7B expression has been revealed.

Our results showed that GAGE7B was significantly upregulated in metastatic tissues. GAGE7B mRNA expression was also upregulated in samples from advanced-stage cancer (stages III/IV) compared with samples of early-stage cancer (stages I/II). GAGE7B protein expression was undetectable by IHC in non-tumorous gastric mucosa, in accordance with previous reports in which no expression of GAGE protein was found in normal tissues [5, 6]. The histoscore of GAGE7B protein expression was higher in metastatic gastric cancer tissues than that in nonmetastatic tissues and was further increased in metastatic loci compared with paired primary tumors. Our results also showed that GAGE7B might play an important role in the initiation and progression of gastric cancer. Patients with high GAGE7B expression in tumors had a shorter OS and DFS time. This is consistent with previous studies showing that the expression levels of GAGE genes are correlated with poor prognoses in neuroblastoma, esophageal carcinoma, and an intestinal type of gastric cancer [7–9]. Results of this study suggest that GAGE7B could be unfavorable markers in gastric cancer patients.

Next, the effect of GAGE7B on the biological behavior of gastric cancer cells was explored. Increased migration, invasion, and distant metastatic ability were observed with GAGE7B overexpression both in vitro and in vivo. In contrast, knockdown of GAGE7B decreased the metastatic capability of gastric cancer cells. Although the effect of GAGE7B on the proliferation of cancer cells was not observed in vitro, tumor growth was significantly increased with GAGE7B overexpression in vivo. Compared with the tumor environment in vitro, the tumor environment in vivo is more similar to that in the human body. We found that although GAGE7B overexpression did not affect the proliferation of cancer cells in vitro, but it promoted tumor growth in nude mice, implying that tumor environment related factor may be involved the tumor growth mediated by GAGE7B overexpression in vivo.

Tumor angiogenesis is essential for tumor growth and metastasis as it supplies the tumor with oxygen and nutrients and may also be a channel for hematogenous metastasis of cancer cells. The results of the present study revealed that GAGE7B stimulates tumor angiogenesis in gastric cancer, suggesting that GAGE7B contributes to the growth and metastasis of gastric cancer, at least partially, by stimulating tumor angiogenesis.

Consistent with our findings, in a previous study, GAGE12B (also known as GAGE12D) was reported to mediate gastric cancer metastasis and growth [11]. Therefore, the results of our study along with that study’s results may suggest that different members of the GAGE family have identical pro-oncogenic roles in gastric cancer.

The current study examined the mechanism underlying the role of GAGE7B in gastric cancer. The pathways related to tumor metastasis and growth were selected for further analysis in an mRNA microarray assay, and the p38δ/pMAPKAPK2/pHSP27 pathway was found to be activated in GAGE7B-overexpressing cells. The increased expression levels of p38δ, pMAPKAPK2, and pHSP27 were further validated in gastric cancer cells overexpressing GAGE7B. p38MAPKs consist of four isoforms, p38α, p38β, p38γ, and p38δ, which are involved in cancer development [18–21]. It has been reported that p38δ promotes the migration, invasion, and proliferation of human cancer cells [22, 23]. It has also been reported that the p38/pMAPKAPK2/pHSP27 pathway is involved in bladder cancer invasion [24]. p38 was also involved in tumor angiogenesis [25]. In previous studies, HSP27, which mediates endothelial cell migration, was found to be the downstream target of p38 [19, 26]. Thus, the results of the current study indicate that GAGE7B-induced p38δ/pMAPKAPK2/pHSP27 pathway, can enhance the metastasis proliferation and angiogenesis of gastric cancer.

Promoter demethylation has been reported to be the underlying mechanism for GAGE family expression [27]. In this study, a novel regulator, miR-30c, was identified and shown to repress GAGE7B expression in gastric cancer. The expression of GAGE7B was dramatically suppressed by miR-30c as demonstrated by dual-luciferase reporter assay and western blot assay. Furthermore, miR-30c expression was found to be inversely correlated with GAGE7B expression in human gastric cancers, further supporting the finding that GAGE7B is the target gene of miR-30c. In addition, in contrast with the good prognosis indicated by low expression of GAGE7B, low expression of miR-30c is associated with poor survival of patients. Moreover, p38δ was also demonstrated to be a target gene of miR-30c. To the best of our knowledge, this is the first study to explore the function of miR-30c in gastric cancer cells. The results suggest that miR-30c can significantly inhibit the migration and invasion ability of gastric cancer cells via inactivating the GAGE7B-induced p38δ/pMAPKAPK2/pHSP27 pathway by directly suppressing GAGE7B and p38δ expression.

It has been reported recently that protein-coding RNAs (mRNAs), and not just non-coding RNAs, can act as ceRNAs by competing specifically for shared miRNAs [17, 28]. Given that GAGE7B and p38δ shared miR-30c in this study, we hypothesized that GAGE7B mRNA could be a ceRNA for p38δ. Our results indicated that GAGE7B-3’UTR, containing a miR-30c binding site, induced p38δ expression and activated the p38δ/pMAPKAPK2/pHSP27 pathway in gastric cancer cells. In addition, an AGO2-RIP assay showed that GAGE7B-3’UTR and p38δ-3’UTR-1 were enriched in RISC, which is necessary for miRNA-mRNA interactions. Although the activity of ceRNAs can be influenced by some factors, such as ceRNA abundance, miRNA abundance, and subcellular localization [29, 30], our results indicate that GAGE7B functions as a ceRNA for p38δ by another mechanism, sequestering miR-30c.

## Conclusions

**Fig. 6:**
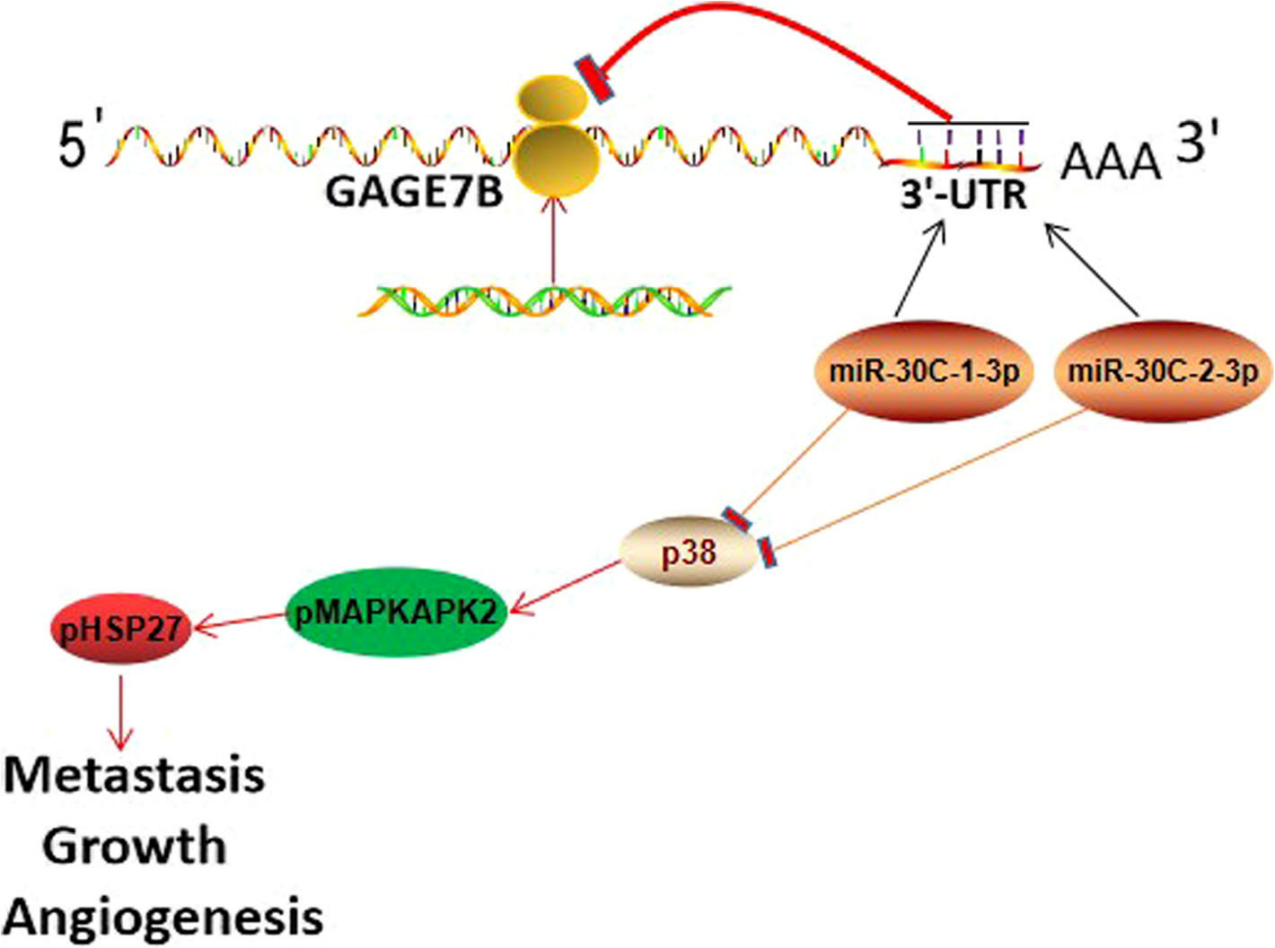
GAGE7B, acted as a ceRNA for p38δ by competing for miR-30c, promoted tumor metastasis, growth and angiogenesis in gastric cancers by activating p38δ/ pMAPKAPK2/ pHSP27 pathway

This study is the first to indicate that the expression of GAGE7B is increased in metastatic gastric cancers and is associated with poor OS and DFS in patients. GAGE7B significantly promotes gastric cancer metastasis, growth, and angiogenesis by upregulating the p38δ/pMAPKAPK2/pHSP27 pathway. The accumulation of GAGE7B can be attenuated by miR-30c in gastric cancer, and GAGE7B is a ceRNA for p38δ which acts by competing for shared miR-30 (Fig. 6). GAGE7B or miR-30c might be potential therapeutic targets for gastric cancer.

## Additional file

Additional file 1: **Figure S1.** GAGE7B enhanced the migration and invasion ability of gastric cancer cells. **Figure S2.** GAGE7B was successfully overexpressed in gastric cancer cells. **Figure S3.** Overexpression of GAGE7B had no effect on gastric cancer cells’ proliferation ability in vitro. **Figure S4.** Downregulation of GAGE7B could not influence the proliferation ability of gastric cancer cells in vitro. **Figure S5.** The activity of p38δ/ pMAPKAPK2/ pHSP27 and PI3K/AKT pathways were enhanced by GAGE7B in gastric cancer. **Figure S6.** The expression of GAGE7B was associated with tumor angiogenesis. **Figure S7.** GAGE7B and p38δ (MAPK13) were negatively regulated by miR-30C. **Figure S8.** The expression of miR-30C was successfully overexpressed and inhibited respectively in MKN45 and BGC823 cells. **Figure S9.** The proliferation ability of gastric cancer cells could not be affected upon miR-30C overexpression in vitro. **Figure S10.** The proliferation ability of gastric cancer cells could not be influenced upon miR-30c downregulation in vitro. **Table S1.** Multivariate analysis of DFS and OS of 132 patients with gastric cancer. **Table S2.** Clinicopathologic characteristics of gastric cancers associated with miR-30c-1-3p and miR-30c-2-3p expression. **Table S3.** Sequences of RT-qPCR primers and siRNA. (DOCX 2419 kb)

## Abbreviations

ceRNA: Competing endogenous RNA
CTA: Cancer testis antigen
GAGE7B: G antigen 7B
HSP27: Heat shock protein family 27
IHC: Immunohistochemical
LNM: Lymph node metastasis
RT-qPCR: Real-time quantitative PCR
