# Supplementary material for: GAGE7B promotes tumor metastasis and growth via activating the p38δ/pMAPKAPK2/pHSP27 pathway in gastric cancer
Source: J Exp Clin Cancer Res. 2019 Mar 11;38:124. doi: 10.1186/s13046-019-1125-z (PMC6419436; doi:10.1186/s13046-019-1125-z)
Supplement: Supplementary file 1 — Figure S1. GAGE7B enhanced the migration and invasion ability of gastric cancer cells. Figure S2. GAGE7B was successfully overexpressed in gastric cancer cells. Figure S3. Overexpression of GAGE7B had no effect on gastric cancer cells’ proliferation ability in vitro. Figure S4. Downregulation of GAGE7B could not influence the proliferation ability of gastric cancer cells in vitro. Figure S5. The activity of p38δ/ pMAPKAPK2/ pHSP27 and PI3K/AKT pathways were enhanced by GAGE7B in gastric cancer. Figure S6. The expression of GAGE7B was associated with tumor angiogenesis. Figure S7. GAGE7B and p38δ (MAPK13) were negatively regulated by miR-30C. Figure S8. The expression of miR-30C was successfully overexpressed and inhibited respectively in MKN45 and BGC823 cells. Figure S9. The proliferation ability of gastric cancer cells could not be affected upon miR-30C overexpression in vitro. Figure S10. The proliferation ability of gastric cancer cells could not be influenced upon miR-30c downregulation in vitro. Table S1. Multivariate analysis of DFS and OS of 132 patients with gastric cancer. Table S2. Clinicopathologic characteristics of gastric cancers associated with miR-30c-1-3p and miR-30c-2-3p expression. Table S3. Sequences of RT-qPCR primers and siRNA. (DOCX 2419 kb) [file 13046_2019_1125_MOESM1_ESM.docx]

**Supplementary**

**Additional file, Figure S1**


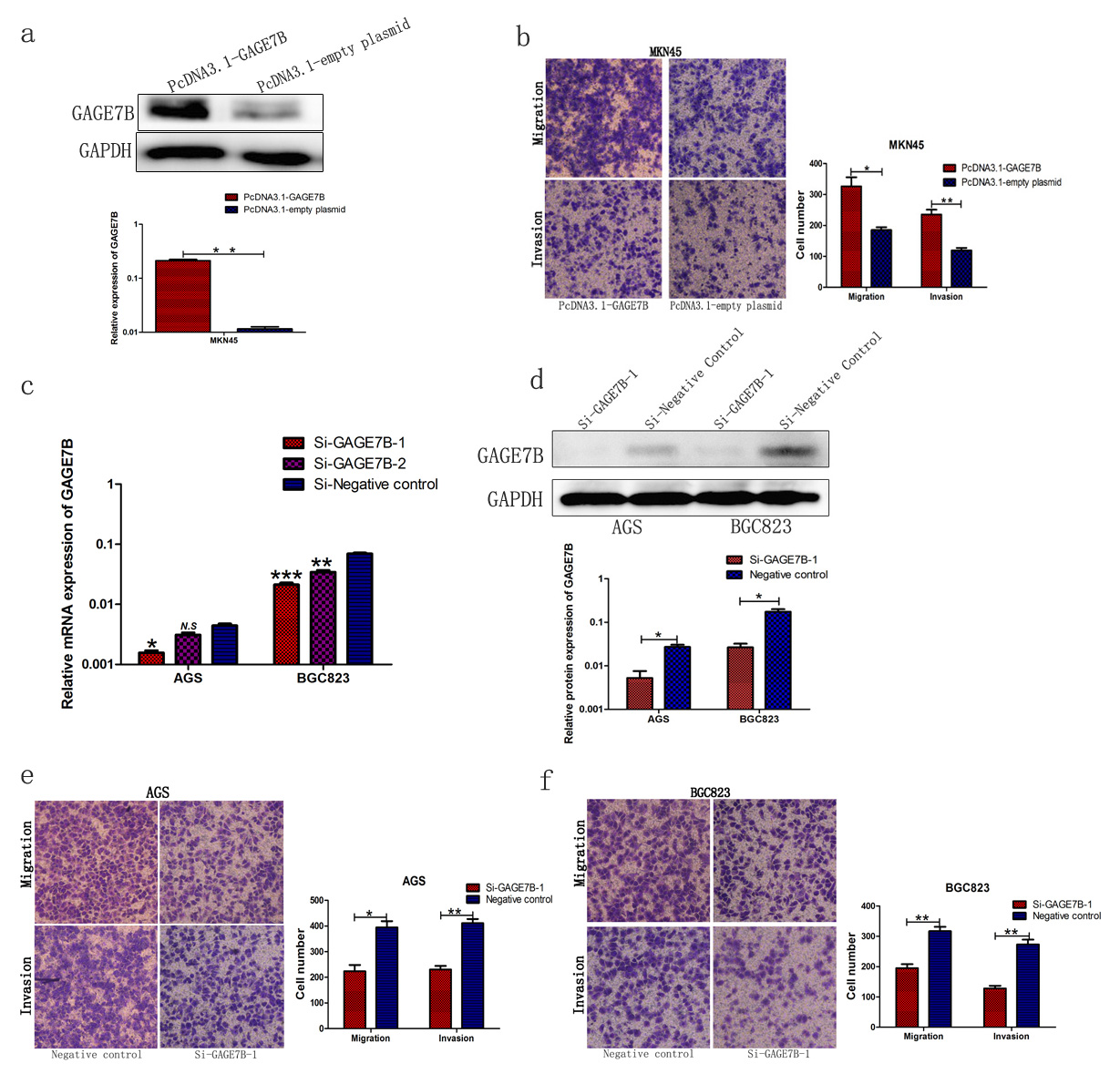


**Additional file, Figure S1: GAGE7B enhanced the migration and invasion ability of gastric cancer cells.**

a and b. The MKN45 cells were transfected with PcDNA3.1-GAGE7B or PcDNA3.1-empty plasmid, the results of Western-blot showed that GAGE7B was successfully overexpressed in MKN45 cells (a), and the migration and invasion ability of MKN45 cells was significantly enhanced by GAGE7B (b) (*t-test, **P < 0.01, *P < 0.05*).

c-f. AGS and BGC823 cells were transfected with siRNA (si-GAGE7B-1 and si-GAGE7B-2) against GAGE7B, or negative control, the result suggested that si-GAGE7B-1 decreased GAGE7B mRNA expression dramatically in gastric cancer cells and the knockdown of GAGE7B by si-GAGE7B-1 was more significant than that by si-GAGE7B-2 (c) detected by RT-qPCR. Therefore, si-GAGE7B-1 was selected for further study. In addition, the protein expression of GAGE7B were dramatically downregulated by si-GAGE7B-1 in gastric cancer cells in Western-blot assay (d). (*t-test, ***P < 0.001, *P < 0.05*). The migration and invasion ability of AGS and BGC823 cells was significantly reduced after the downregulation of GAGE7B (e and f). (*t-test, **P < 0.01, *P < 0.05*).

**Additional file, Figure S2**


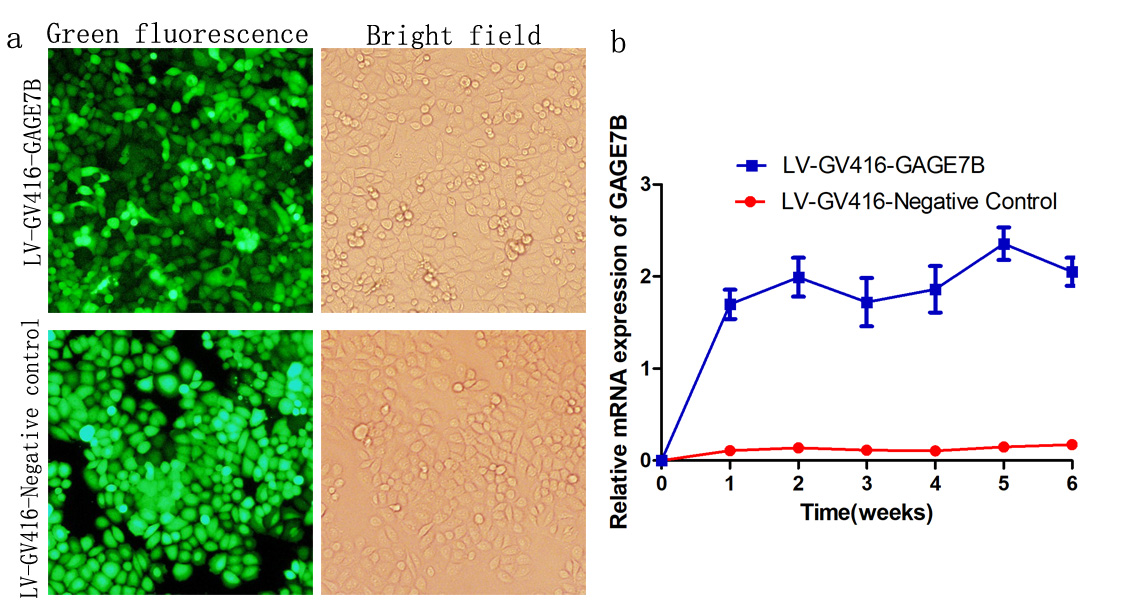


**Additional file, Figure S2. GAGE7B was successfully overexpressed in gastric cancer cells.**

The gastric cancer cells BGC823 were transfected with LV-GV416-GAGE7B and LV-GV416-Negative control with GFP as mark protein. The transfected cells successfully expressed mark protein GFP ( × 100) (a) and the GAGE7B was stably overexpressed in gastric cancer cells during 6 weeks of the experiment examined by RT-qPCR, compared with negative control cells (*t-test, **P < 0.01*) (b).

**Additional file, Figure S3.**

**
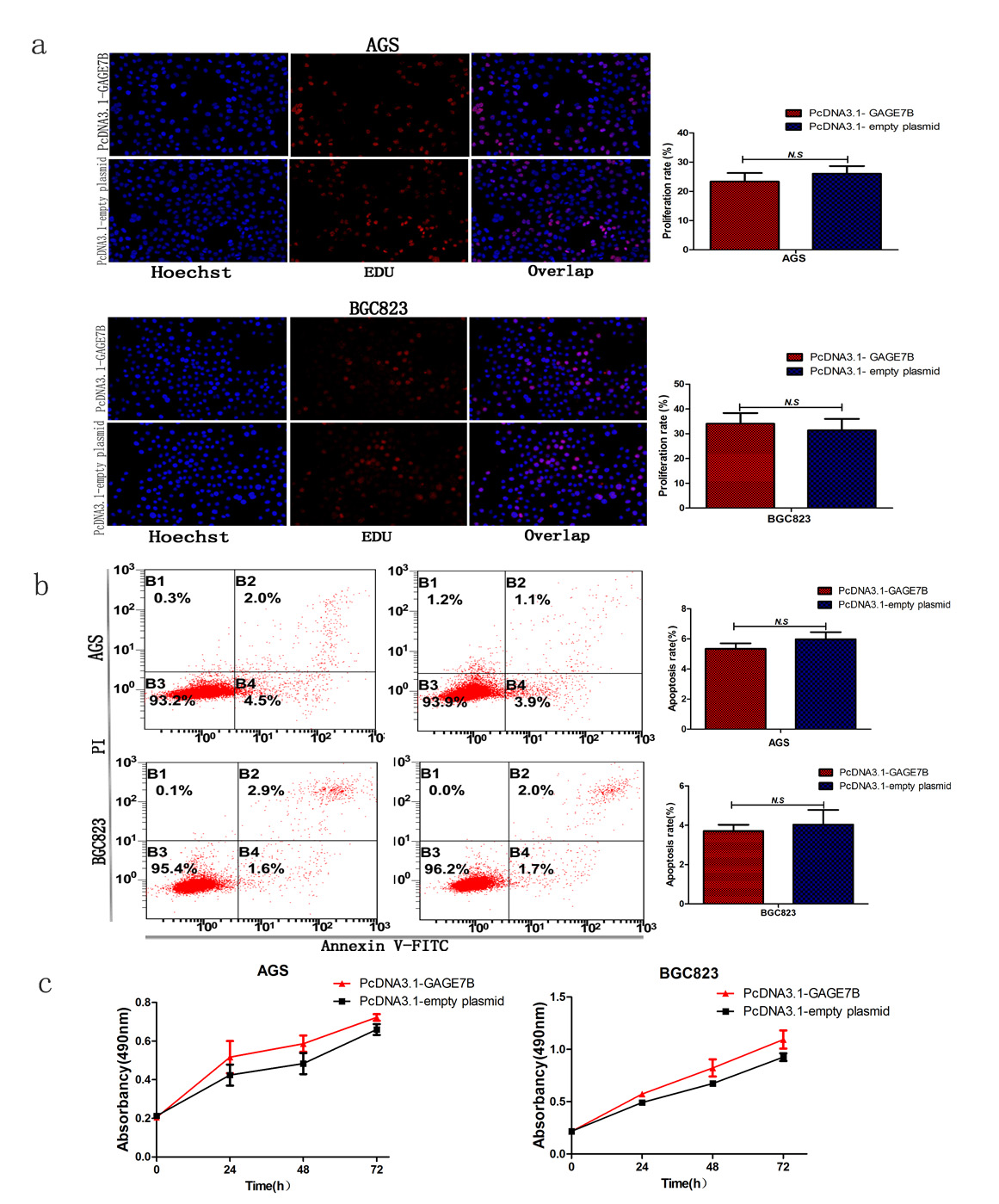
**

**Additional file, Figure S3. Overexpression of GAGE7B had no effect on gastric cancer cells’ proliferation ability in *vitro*.**

The AGS and BGC823 cells were first transfected with PcDNA3.1-GAGE7B or PcDNA3.1-empty plasmid, then the proliferation and apoptosis ability of the cells were investigated by EDU, flow cytometry and MTS assays. The results of EDU assay suggested that there was no significant difference of proliferate rate between the cells overexpressing GAGE7B and the negative control cells (a and b). GAGE7B had no significant influence on gastric cancer cells’ apoptosis rate in flow cytometry assay (c). Consistently, no significant effect on the proliferation ability of AGS and BGC823 cells was observed between the GAGE7B group and negative control group in MTS assay (d and e). (*t-test, P > 0.05*).

**Additional file, Figure S4.**

**
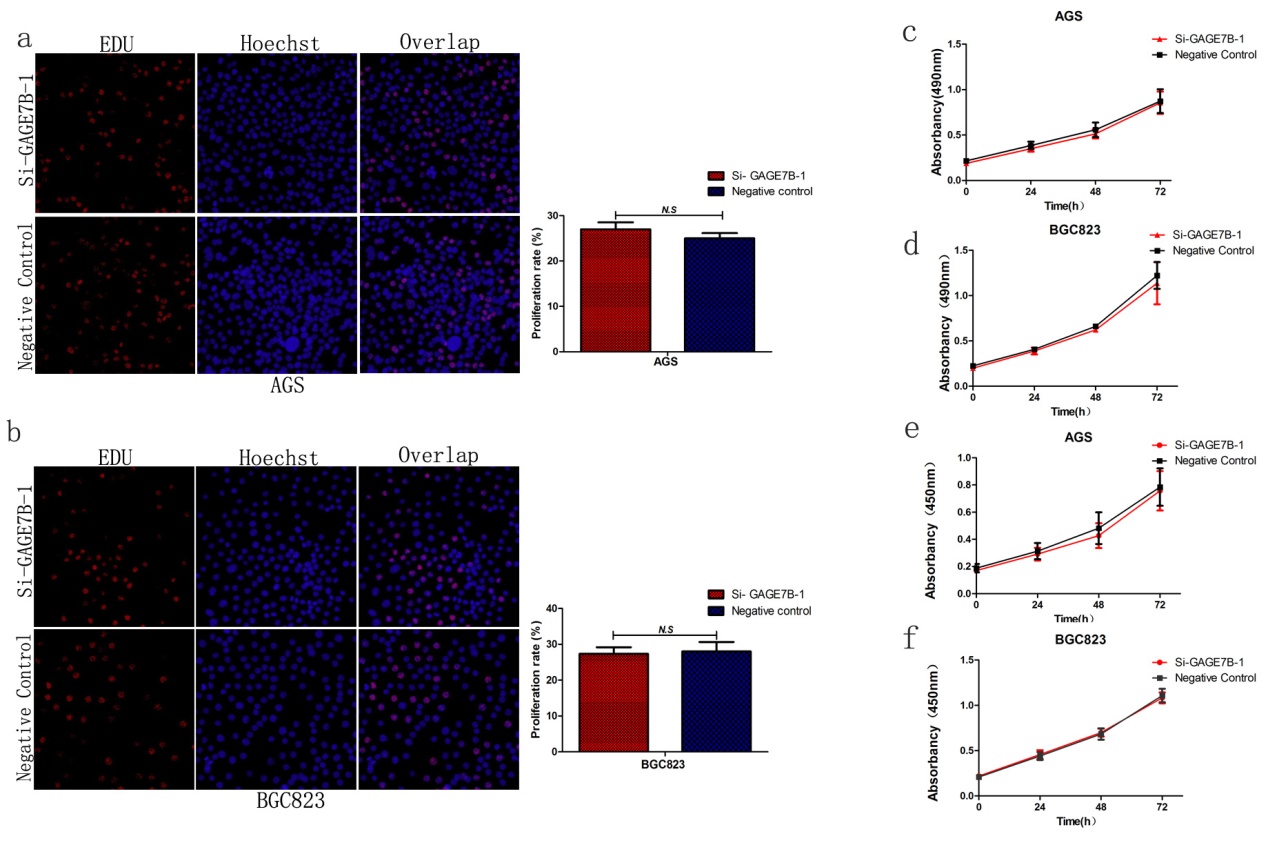
**

**Additional file, Figure S4. Downregulation of GAGE7B could not influence the proliferation ability of gastric cancer cells *in vitro*.**

The GAGE7B expression in AGS and BGC823 cells was downregulated by si-GAGE7B-1, then the influence on the cells’ proliferation ability was detected in *vitro.* The data indicated that downregulation of GAGE7B could not influence the proliferation of AGS and BGC823 cells detected by EDU (a and b), MTS (c and d) and CCK8 (e and f) assays. (*t-test, P > 0.05*).

**Additional file, Figure S5**


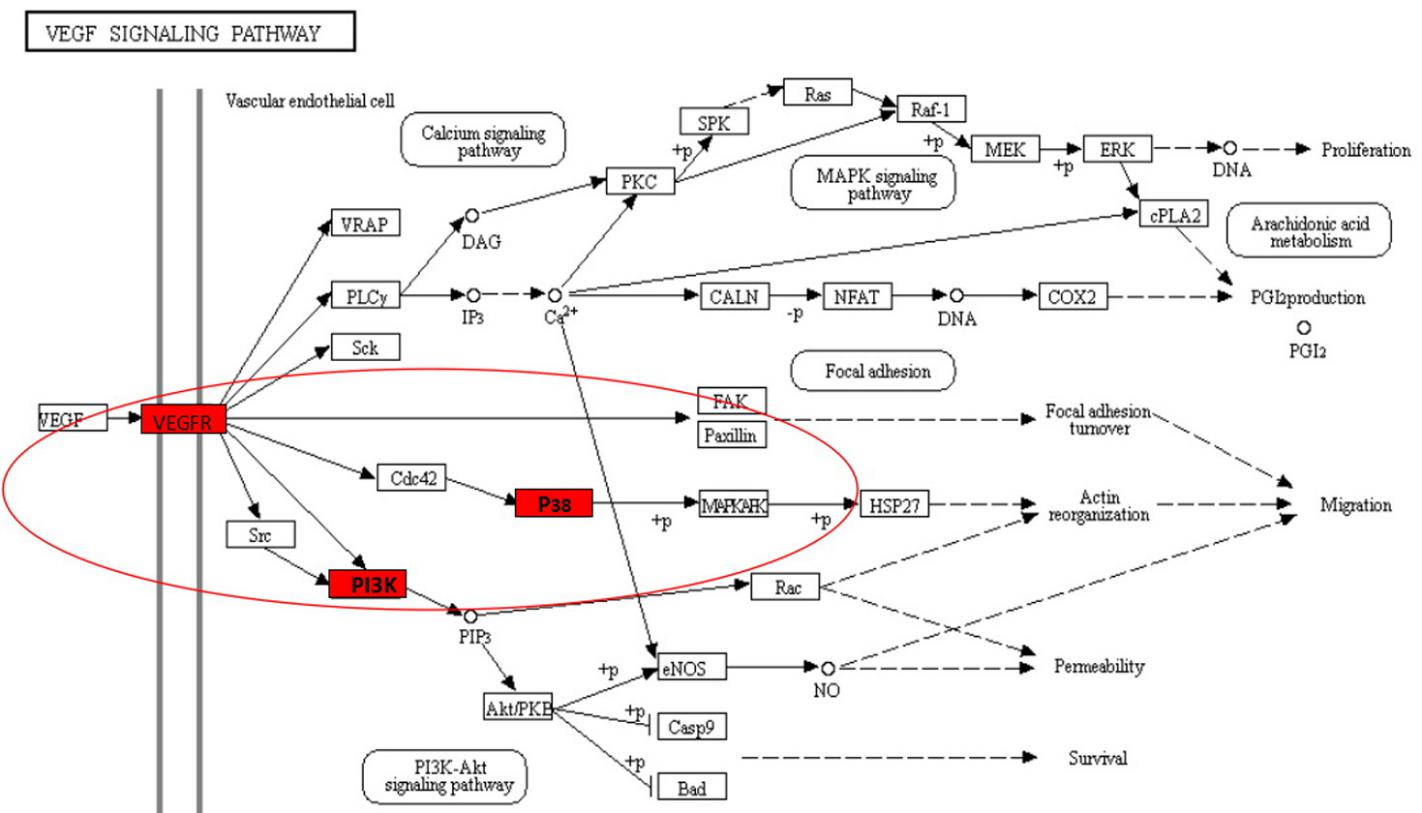


**Additional file, Figure S5: The activity of p38δ/ pMAPKAPK2/ pHSP27 and PI3K/AKT pathways were enhanced by GAGE7B in gastric cancer.**

The BGC823 cells were transfected with PcDNA3.1-GAGE7B or PcDNA3.1-empty plasmid then were subjected to mRNA microarray assay. The differentially expressed pathways that related to tumor progression were selected for further analysis. The results showed that the activity of p38δ/pMAPKAPK2/pHSP27 and PI3K/AKT pathways were enhanced by GAGE7B, as the expressions of p38 and PI3K were upregulated (≧2.0 fold) in microarray assay.

**Additional file, Figure S6**

**
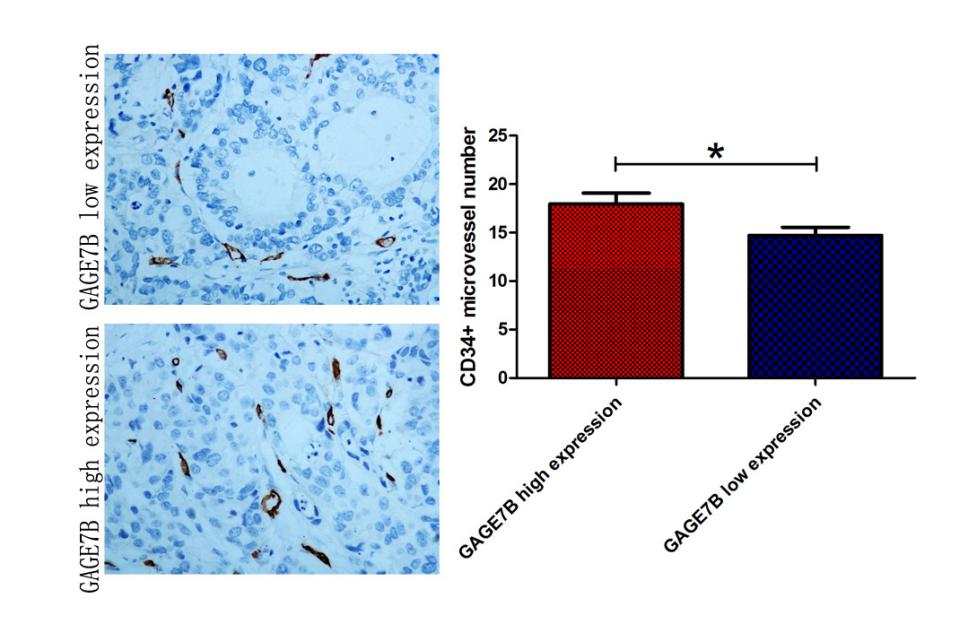
**

**Additional file, Figure S6. The expression of GAGE7B was associated with tumor angiogenesis.**

IHC was performed for CD34 staining in 132 cases of gastric cancer tissues. The CD34+ microvessel number was counted in each case and was further analyzed between GAGE7B low expression group and GAGE7B high expression group ( × 400). The GAGE7B high group showed more number CD34+ microvessels than that in GAGE7B low expression group, which suggested that the CD34+ microvessel number was positively associated with GAGE7B expression in gastric cancer (*t-test, *P < 0.05*).

**Additional file, Figure S7**

**
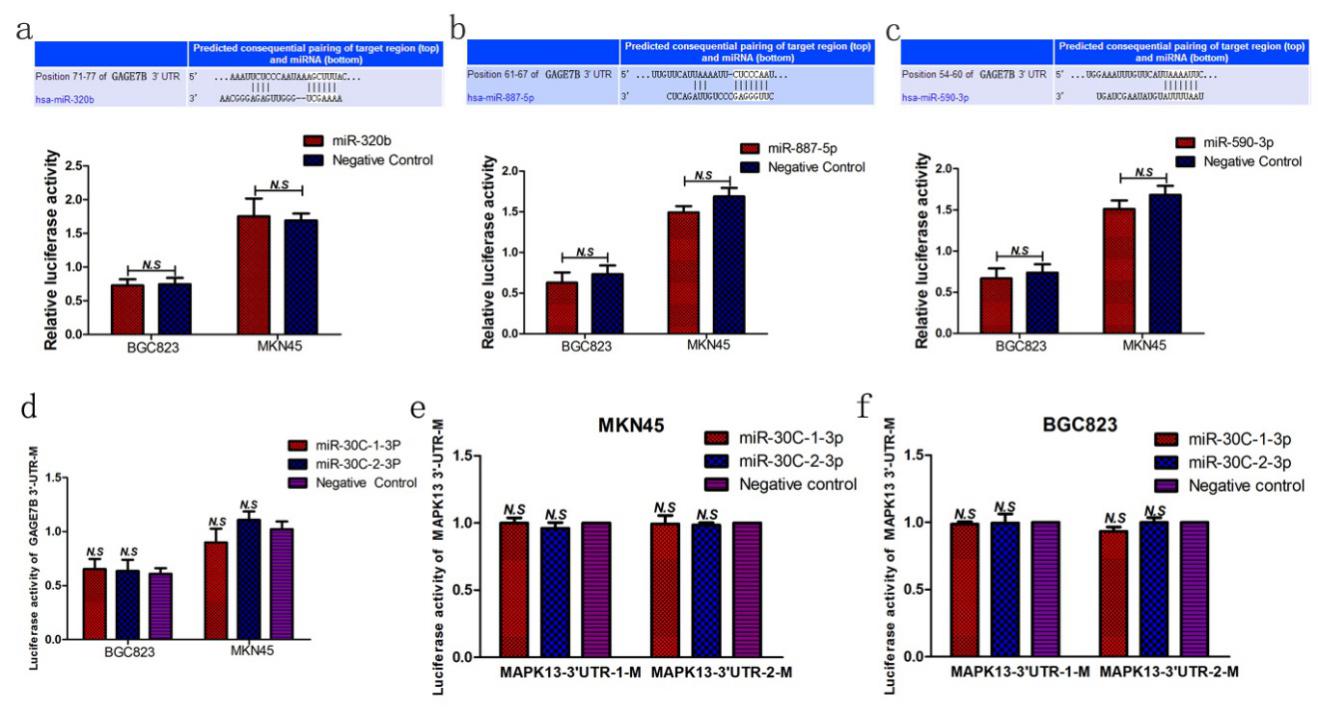
**

**Additional file, Figure S7. GAGE7B and p38**δ **(MAPK13) were negatively regulated by miR-30C.**

a-c. The miRNAs that could negatively regulate GAGE7B were predicted by software and then dual-luciferase reporter assay was performed to identify the target miRNAs. The prediction showed miR-320b (a), miR-887-5p (b) and miR-590-3p (c) potentially bind to the 3’-UTR region of GAGE7B, however, the results of dual-luciferase reporter assay suggested that the luciferase activity of GAGE7B pmirGLO-3’ UTR vector was not significantly influenced by the above three miRNAs. (*t-test, P > 0.05*)

d. When the binding site of miR-30c in the GAGE7B-3’UTR was mutated, the repression of luciferase activity by miR-30c was attenuated in duel-luciferase reporter assay. (*t-test, P > 0.05*)

e and f. When the two binding sites of miR-30c in the p38δ (MAPK13)-3’UTR were mutated respectively, no significant influence on the luciferase activity was detected between the cells transfected with miR-30c and the cells of negative control group (e and f). (*t-test, P > 0.05*).

**Additional file, Figure S8**


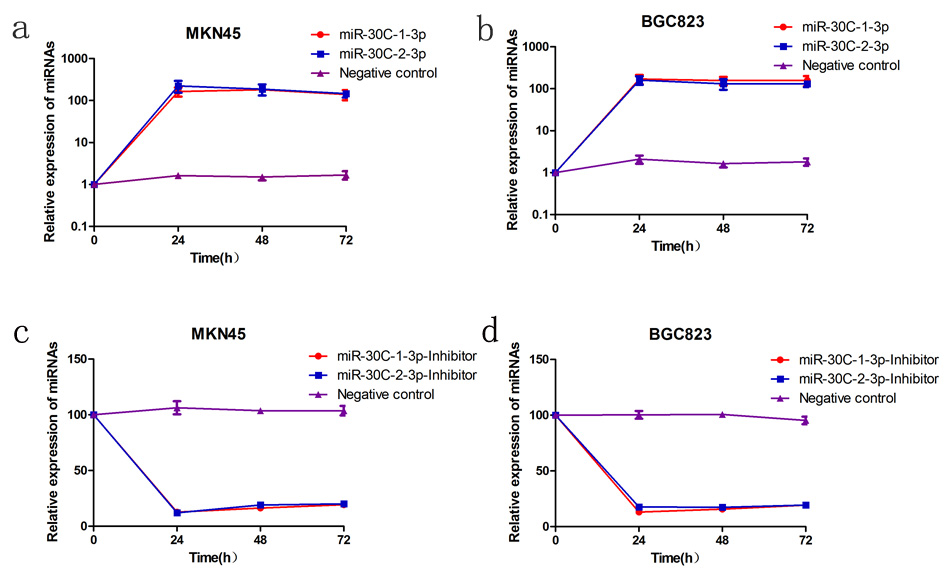


**Additional file, Figure S8. The expression of miR-30C was successfully overexpressed and inhibited respectively in MKN45 and BGC823 cells.**

a and b. The expressions of miR-30c-1-3p and miR-30c-2-3p in AGS and BGC823 cells were examined 24, 48 and 72 hours after the transfection, the results indicated that miR-30c-1-3p and miR-30c-2-3p were successfully overexpressed in gastric cancer cells.

c and d. The expressions of miR-30c-1-3p and miR-30c-2-3p in MKN45 and BGC823 cells were dramatically downregulated, 24, 48 and 72 hours after the transfection of miR-30c inhibitors.

**Additional file, Figure S9.**

**
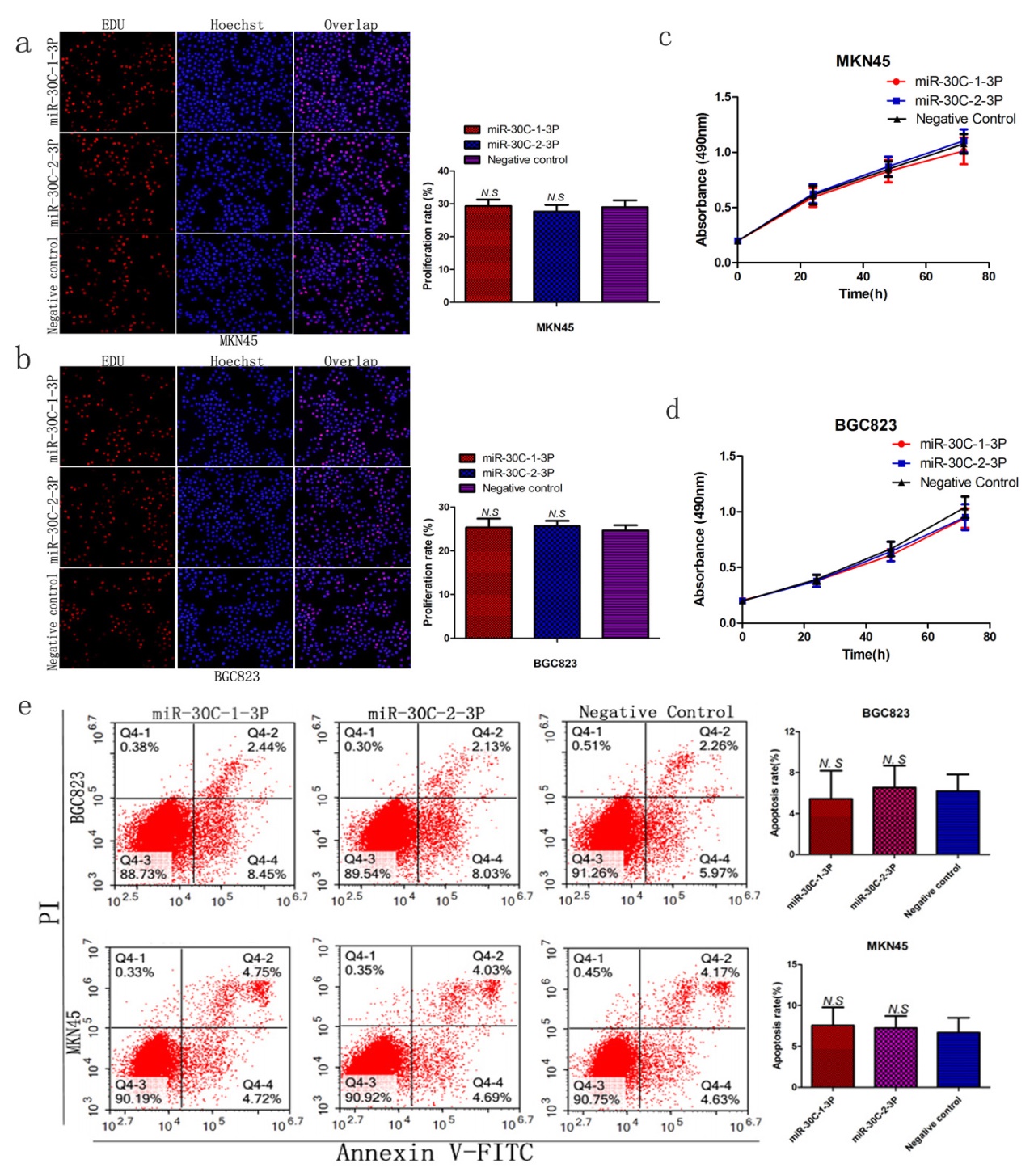
**

**Additional file, Figure S9. The proliferation ability of gastric cancer cells could not be affected upon miR-30C overexpression in *vitro*.**

The proliferation and apoptosis ability of MKN45 and BGC823 cells was investigated upon overexpression of miR-30c-1-3p and miR-30c-2-3p. The results suggested that the proliferation and apoptosis ability of MKN45 and BGC823 cells could not be affected by miR-30c-1-3p and miR-30c-2-3p in EDU (a and b), MTS (c and d) and flow cytometry (e ) assays. (*t-test, P > 0.05*).

**Additional file, Figure S10**


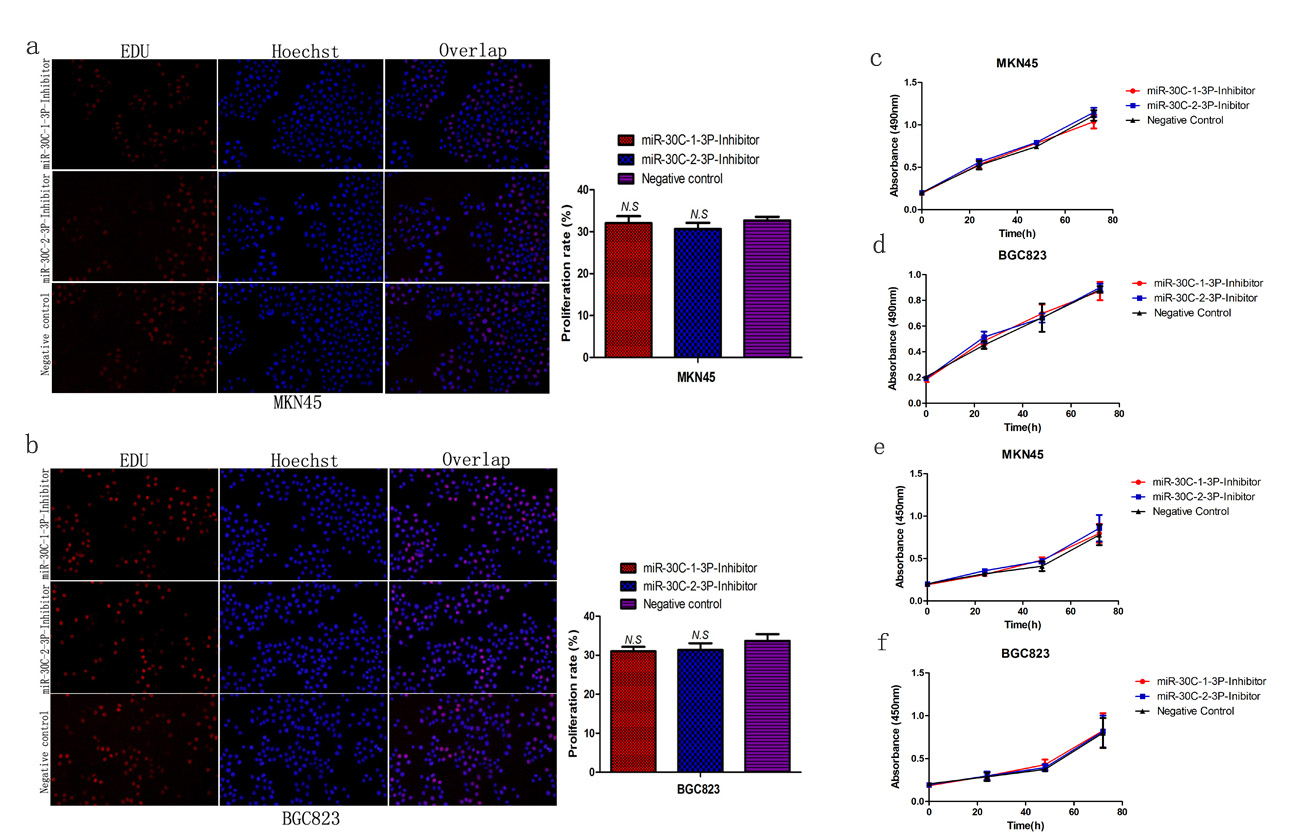


**Additional file, Figure S10. The proliferation ability of gastric cancer cells could not be influenced upon miR-30c downregulation in *vitro*.**

a and b. Knockdown of miR-30c-1-3p and miR-30c-2-3p did not affect the proliferation ability of MKN45 (a) and BGC823 (b) cells significantly, compared with that in negative control group. (*t-test, P > 0.05*).

c-f. Upon knockdown of miR-30c-1-3p and miR-30c-2-3p in MKN45 and BGC823, the cells’ proliferation and apoptosis ability was detected and no significant influence of miR-30c-1-3p and miR-30c-2-3p was found in MTS (c and d) and CCK8 assays (e and f). (*t-test, P > 0.05*).

| **Additional file, Table S1: Multivariate analysis of DFS and OS of 132 patients with gastric cancer** | | | | | |  |
| --- | --- | --- | --- | --- | --- | --- |
|  | | | | | | |
|  | | **Disease Free Survival** |  |  | **Overall Survival** |  |
| **Variables** | | **HR (95% CI)** | ***P*** |  | **HR (95% CI)** | ***P*** |
| **Clinical stage** | |  |  |  |  |  |
| I / II | | 3.825 | ***< 0.001*** |  | 2.937 | ***0.001*** |
| III / IV | | (1.873 - 7.814) |  |  | (1.578 - 5.465) |  |
| **Lymph node metastasis** | |  |  |  |  |  |
| Yes | | 1.035 | *0.904* |  | 1.369 | *0.267* |
| No | | (0.589 - 1.819) |  |  | (0.786 -2.385) |  |
| **Tumor size** | |  |  |  |  |  |
| < 5 cm | | 1.745 | ***0.027*** |  | 1.693 | ***0.024*** |
| ≧ 5cm | | (1.065 - 2.860) |  |  | (1.073 - 2.672) |  |
| **Lauren’s classification** | |  |  |  |  |  |
| Intestinal type | | 1.206 | *0.476* |  | 1.254 | *0.350* |
| Diffuse type | | (0.720 - 2.018) |  |  | (0.781 - 2.013) |  |
| **GAGE7B expression** | |  |  |  |  |  |
| Low | | 1.351 | *0.242* |  | 1.109 | *0.665* |
| High | | (0.816-2.237) |  |  | ( 0.694 - 1.771) |  |

**Additional file, Table S2: Clinicopathologic characteristics of gastric cancers associated with miR-30c-1-3p and miR-30c-2-3p expression**

|  |  | **miR-30c-1-3p** | |  | | **miR-30C-2-3p** | |
| --- | --- | --- | --- | --- | --- | --- | --- |
| **Variables** | **No.** | **Median** | **P-value** | | **Median** | | **P-value** |
| **Age (y)** |  |  |  |  | |  |  |
| ≤ 60 | 42 | 0.0117 | 0.033 |  | | 0.0033 | 0.020 |
| ﹥ 60 | 44 | 0.0050 |  |  | | 0.0016 |  |
| **Gender** |  |  |  |  | |  |  |
| Male | 75 | 0.0054 | 0.179 |  | | 0.0018 | 0.103 |
| Female | 11 | 0.0156 |  |  | | 0.0093 |  |
| **Tumour size (mm)** |  |  |  |  | |  |  |
| ＜50 | 45 | 0.0105 | 0.272 |  | | 0.0030 | 0.078 |
| ≥ 50 | 41 | 0.0050 |  |  | | 0.0015 |  |
| **Clinical stage** |  |  |  |  | |  |  |
| I / II | 29 | 0.0086 | 0.121 |  | | 0.0026 | 0.366 |
| III /IV | 57 | 0.0052 |  |  | | 0.0017 |  |
| **Lymph node metastasis** |  |  |  |  | |  |  |
| Yes | 61 | 0.0105 | 0.020 |  | | 0.0018 | 0.815 |
| No | 25 | 0.0027 |  |  | | 0.0024 |  |
| **WHO histological classification** |  |  |  |  | |  |  |
| Well-Moderately differentiated | 25 | 0.0054 | 0.029 |  | | 0.0018 | 0.318 |
|  |  |  |  |  | |  |  |
| Poorly differentiated | 61 | 0.0064 |  |  | | 0.0019 |  |
|  |  |  |  |  | |  |  |
| **Lauren’s classification** |  |  |  |  | |  |  |
| Intestinal type | 37 | 0.0069 | 0.827 |  | | 0.0019 | 0.222 |
| Diffuse type | 49 | 0.0050 |  |  | | 0.0018 |  |
|  |  |  |  |  | |  |  |

| **Additional file, TableS 3. Sequences of RT-qPCR primers and siRNA** | |
| --- | --- |
| **Gene** | **Sequence** |
| GAGE7B -F | 5’-GGGAACCAGCAACTCAACG -3’ |
| GAGE7B -R | 5’-CCCATCAGGACCATCTTCAC -3’ |
| PHGR1-F | 5’-AGATGGCAAGCCTGAGAGAA-3’ |
| PHGR1-R | 5’-AGAATAGAATGTGGCCTCTAGGA-3’ |
| ZNF503-F | 5’-CCCCTACGGCTTTATGCTCC-3’ |
| ZNF503-R | 5’-GGCCAGAGACGACGAGC-3’ |
| GAGE7B-3’UTR-F | 5’-CAGGCTGCTCCTATGTTGGAA-3’ |
| GAGE7B-3’UTR-R | 5’-TCTTTGCAGAAGGCTGTAAAGC-3’ |
| p38δ-3’UTR-1-F | 5’-AACAGCATGACACACATAGCC-3’ |
| p38δ-3’UTR-1-R | 5’-GGGAGAGGCAATATCGGGAC-3’ |
|  |  |
| Si-GAGE7B-1 | 5’- GGAGCAUCUGCAGGUCAAGTT-3’  5’- CUUGACCUGCAGAUGCUCCTT -3’ |
| Si-GAGE7B-2 | 5’- CCUGAAAUGAUUGGGCCUATT-3’ 5’-UAGGCCCAAUCAUUUCAGGTT-3’ |
|  |  |
|  |  |
|  |  |
|  |  |
|  |  |
|  |  |
|  |  |
|  |  |
|  |  |
